# Pregnancy complications and risk of uterine rupture among women with singleton pregnancies in China

**DOI:** 10.1186/s12884-022-04465-w

**Published:** 2022-02-16

**Authors:** Jing Tao, Yi Mu, Peiran Chen, Yanxia Xie, Juan Liang, Jun Zhu

**Affiliations:** 1 National Office for Maternal and Child Health Surveillance of China, West China Second University Hospital, Sichuan University, Chengdu, Sichuan China; 2 Key Laboratory of Birth Defects and Related Diseases of Women and Children (Sichuan University), Ministry of Education, Chengdu, China

**Keywords:** Pregnancy complications, Uterine rupture, Risk factors, Large for gestational age, Preterm delivery

## Abstract

**Background:**

The goal of this study was to investigate whether pregnancy complications are associated with an increased risk of uterine rupture (UR) and how that risk changes with gestational age.

**Methods:**

We obtained all data from China’s National Maternal Near Miss Surveillance System (NMNMSS) between 2012 and 2018. Poisson regression analysis was used to assess the risk of UR with pregnancy complications (preeclampsia, gestational diabetes mellitus, placental abruption, placenta previa and placenta percreta) among 9,454,239 pregnant women. Furthermore, we analysed the risks of UR with pregnancy complications in different gestational age groups.

**Results:**

The risk of UR was increased 2.0-fold (1.2-fold to 2.7-fold) in women with pregnancy complications (except for preeclampsia). These associations also persisted in women without a previous caesarean delivery. Moreover, an increased risk of UR before term birth was observed among women with gestational diabetes mellitus, placental abruption and placenta percreta. The risk of UR was slightly higher in women with gestational diabetes mellitus who had a large for gestational age (LGA) foetus, especially at 32 to 36 weeks gestation.

**Conclusions:**

The risk of UR is associated with gestational diabetes mellitus, placental abruption, placenta previa and placenta percreta, but varies in different gestational ages.

**Supplementary Information:**

The online version contains supplementary material available at 10.1186/s12884-022-04465-w.

## Background

Uterine rupture (UR) is a tear in the uterine wall that occurs before or during labour. It poses considerable risks for adverse maternal and perinatal outcomes, including serious health risks for both mother (e.g., maternal death) and foetus (e.g., stillbirth, neonatal death) [1, 2]. Mounting evidence supports that a history of caesarean section is a major risk factor for UR in subsequent pregnancies [1, 3], and the risk increases with the number of previous cesarean deliveries [4, 5]. The incidence of UR varies across countries, ranging from 0.18 to 9 cases per 1,000 pregnant women [1, 6, 7]. Many countries have created policies to decrease caesarean rates [8, 9], but the UR rate has increased over the years [10, 11]. It is unknown whether the increasing rate of UR is due to the effects of potential risk factors related to a history of caesarean section.

Evidence suggests that women who have a history of caesarean section may be at increased risk of pregnancy complications, such as placental abruption, placenta previa, placenta percreta, gestational diabetes mellitus and preeclampsia [12–14]. These pregnancy complications may be partly considered manifestations of dysfunctional placental function [15, 16]. To date, placenta percreta has been reported to be associated with an increased risk of UR [17, 18]. However, there are limited data on the association of other pregnancy complications with UR. A few studies with small sample sizes have analysed the risk of UR with diabetes and hypertension [7, 19, 20], but these studies have yielded different conclusions. In addition, current guidelines only discuss the impact of vaginal trial delivery after caesarean section on the occurrence of UR [21, 22], but the effects of pregnancy complications related to previous caesarean deliveries have not been reported. If a link between pregnancy complications and UR is confirmed, it may provide additional preventive measures.

We hypothesize that pregnancy complications (preeclampsia, gestational diabetes mellitus, placental abruption, placenta previa, placenta percreta) may be associated with an increased risk of UR. Therefore, our study analysed more than 9 million singleton pregnant women from China’s National Maternal Near Miss Surveillance System (NMNMSS) to evaluate whether these pregnancy complications were associated with the risk of UR. By analysing the impact of each pregnancy complication in different gestational age groups, we provided insights into the early interventions that would contribute to reducing the incidence of UR in pregnant women.

## Methods

### Design and setting

We obtained data from China’s NMNMSS between 2012 and 2018. The system covers 438 hospitals in 326 districts or counties throughout 30 provinces, each of which manages more than 1,000 deliveries annually [11]. Data collected included sociodemographic characteristics, obstetric history, pregnancy complications, and pregnancy outcomes of all pregnant or postpartum women in a hospital. Doctors in each hospital were trained to collect data prospectively from admission to discharge. Quality assurance was ensured by staff from county-level, municipal-level and provincial-level maternal and child health hospitals 1–2 times a year. At the same time, the National Office for Maternal and Child Health Surveillance verified the quality of the records by selecting 6–8 hospitals randomly in each province once a year [23].

### Study population

We restricted the analysis to pregnant women with singleton births who delivered at or after 28 complete weeks of gestation. Women with multiple pregnancies were not included because they are prone to pregnancy complications [24] and UR [25]. Women lacking information on delivery method, history of caesarean section, or gravidity were excluded. We also excluded women with an unlikely combination of gravidity and parity. This left a total of 9,454,239 women for the study.

### Variable definition

UR was defined as uterine or lower uterine dehiscence during late pregnancy or delivery [11]. According to the degree of dehiscence, UR can be divided into complete UR (tearing in all layers of the uterine wall) and incomplete UR (tearing in the muscular layers) [10]. Common clinical manifestations of UR include foetal distress, sudden tearing uterine pain, cessation of uterine contractions and abnormal vaginal bleeding [26]. UR was diagnosed by a health professional with imaging techniques (magnetic resonance imaging or ultrasound examination) [27]; or during emergency caesarean delivery; or peripartum hysterectomy or laparotomy after vaginal birth [3]. Unfortunately, UR is captured as a dichotomous variable (yes/no) in the NMNMSS, and the type of rupture is lacking. We identified five pregnancy complications related to previous caesarean delivery for analysis: preeclampsia, gestational diabetes mellitus, placental abruption, placenta previa and placenta percreta [13, 14]. Preeclampsia included pregnancies with preeclampsia, eclampsia or HELLP (haemolysis, elevated liver enzymes and low platelets) syndrome, as well as chronic hypertension with superimposed preeclampsia. Gestational diabetes was diagnosed by a 2-h 75 g oral glucose tolerance test (OGTT) performs during 24–28 gestational weeks in all pregnant women [28]. Placental abruption was defined as the premature separation of the implanted placenta before delivery. Placenta previa was defined as the placenta covering the internal os of the cervix. Placenta percreta, as the most severe grade of the placenta accreta spectrum disorders, occurs when the chorionic villi penetrate the uterine serosa [29].

We selected variables that may be related to the occurrence of UR, including region, hospital level, education level (none, primary school, middle school, high school, college or higher), maternal age at delivery (< 20, 20–24, 25–29, 30–34, 35–39 and ≥ 40 years), the number of antenatal visits (none, 1–3, 4–6, 7–9, ≥ 10), gravidity (1, 2–3, ≥ 4), parity (0, 1, ≥ 2), number of previous caesarean deliveries (0, 1, ≥ 2), foetal presentation (cephalic and other abnormal lies), gestational age, birthweight, and mode of delivery (vaginal delivery and caesarean section). We divided China’s regions into three categories (eastern, central and western) and classified hospitals into three levels (the first level represents the smallest hospital) according to standard definitions [23]. Gestational age was defined based on ultrasound measurement results or estimated from the date of the last menstrual period and classified as early preterm (28–33 weeks), late preterm (34–36 weeks), or term (≥ 37 weeks). Large for gestational age (LGA) was defined as a gestational age-adjusted birth weight above the 90th percentile [30]. Other factors thought to be associated with UR included gestational hypertension, chronic hypertension, heart disease, hepatic disease, severe anaemia (haemoglobin concentration lower than 70 g/L), infection (excluding abortion-related infection, puerperal infection and abdominal incision infection), thrombophlebitis, renal disease, lung disease, and connective tissue disorders.

### Statistical Analysis

#### Primary analysis

We expressed the UR rate as the number of pregnant women with UR per 1,000 pregnant women. Since some women giving birth in township hospitals were not included in the NMNMSS, we weighted the UR rate for the sampling distribution of the population according to the 2010 census of China, as detailed elsewhere [23]. Moreover, a history of caesarean section is a major risk factor for UR in subsequent pregnancies [1, 3], and the risk increases with the number of previous caesarean deliveries [4, 5]. Thus, we calculated the previous caesarean deliveries adjusted rate of UR in women by using the *margins* command in Stata [31].

We identified five pregnancy complications for analysis: preeclampsia, gestational diabetes mellitus, placental abruption, placenta previa, and placenta percreta. We used Poisson regression with a robust variance estimator to assess the association of UR with pregnancy complications, reporting the results from two models. The reference for each model was women without any of the five pregnancy complications. Model 1 describes the adjusted relative risk (aRR) and 95% confidence Interval (CI), taking into account the sampling distribution of the population and birth clustering within hospitals, medical institutions, and pregnant women's sociodemographic and clinical factors that might contribute to the observed associations. Model 2 adjusted for the covariates in Model 1 as well as the number of previous caesarean deliveries (0, 1, ≥ 2) and LGA (yes/no). We did not adjust for gestational age or final mode of delivery because they included consequences of UR (i.e., laparotomy due to UR). To identify the most robust and stable model, we investigated both multicollinearity and model goodness-of-fit.

Due to a history of caesarean section related to both pregnancy complications [1, 3] and UR [12–14], pregnancy complications may be only an intermediate factor in the causal chain between a history of caesarean section and the risk of UR. We repeated the association analysis of pregnancy complications with UR only in women without previous caesarean delivery.

#### Secondary analysis

##### **Restricting to a group of women**

To investigate the association between pregnancy complications and UR without potential maternal confounding factors (advanced maternal age [1] and multiple gravidities [32]), we performed sensitivity analyses excluding women with advanced maternal age (≥ 35 years) and/or multiple gravidities (≥ 4). Given the possible impacts of abnormal foetal presentation and macrosomia on the occurrence of UR [33], we restricted the association analysis to women with offspring having a cephalic lie and a birth weight of less than or equal to 4000 g.

##### **Co-occurrence of pregnancy complications and UR risk**

Pregnancy complications may co-occur in a given pregnancy. We therefore repeated model 1 and 2 testing for the associations between having at least two or more pregnancy complications and the risk of UR. Because the numbers were too small to assess unique combinations of pregnancy complications, we modelled the variables “no pregnancy complications”, “any one pregnancy complication”, and “any two or more pregnancy complications” in a single model.

##### **Risk of UR in different gestational age groups**

To explore the risk of UR with pregnancy complications in different gestational age groups, we compared the UR rates in women for each pregnancy complication and gestational age against those in women without pregnancy complications at 28–33 weeks of gestational age, using model 1 and 2.

##### **Role of large for gestational age**

Because LGA is associated with gestational diabetes mellitus [34], we repeated model 1 and 2 testing to analyse a possible effect of LGA foetuses on the risk of UR among women with gestational diabetes mellitus.

##### **Trends over time in UR rates**

To examine trends over time in UR rates among women with pregnancy complications, we repeated model 1 and 2 by including the year of study period as a continuous variable.

Statistical analysis was performed using Stata (version 16.0, Stata Corp LP., College Station, United States of America). *P* < 0.05 (2-sided) was considered statistically significant.

##### **Patient involvement**

Informed consent from the patients was waived by the Ethics Committee, as the data used in our study were obtained from a national routine surveillance system established by the government. Data use was authorized by the National Health Commission, and data provided to us were deidentified.

## Results

Of the 9,454,239 pregnant women enrolled in this study, 885,087 (9.4%) women had pregnancy complications. Compared with women without pregnancy complications, women with pregnancy complications tended to be older, to have multiple gravidities and to have had previous caesarean deliveries. At the time of birth, women with pregnancy complications had a higher percentage of abnormal foetal presentations and LGA. Details are summarized in Table 1.

**Table 1:** Maternal and fetal characteristics of 9,454,239 pregnant women with singleton births

Sociodemographic characteristic	Women without pregnancy complications, n (%)	Women with pregnancy complications, n (%)	*P* value
	***n* = 8,569,152 (90.6%)**	***n* = 885,087 (9.4%)**	
**Region of China**			
East	2,407,580 (88.5)	313,912 (11.5)	*P* < 0.001
Central	3,464,042 (91.9)	304,138 (8.1)	
West	2,697,530 (91.0)	267,037 (9.0)	
**Hospital level**			
Unknown	457,421 (95.2)	23,264 (4.8)	*P* < 0.001
Level 1	569,527 (96.8)	18,536 (3.2)	
Level 2	4,094,405 (93.3)	295,839 (6.7)	
Level 3	3,447,799 (86.3)	547,448 (13.7)	
**Maternal education**			
None	42,037 (90.3)	4,523 (9.7)	*P* < 0.001
Primary school	259,703 (91.8)	23,323 (8.2)	
Middle school	2,821,827 (93.9)	182,270 (6.1)	
High school	2,309,079 (90.8)	235,360 (9.2)	
College or higher	2,977,190 (87.9)	409,826 (12.1)	
Unknown	159,316 (84.2)	29,785 (15.8)	
**Mother’s age, years**			
< 20	229,174 (96.5)	8,387 (3.5)	*P* < 0.001
20–24	1,742,770 (95.3)	86,051 (4.7)	
25–29	3,554,091 (92.1)	303,454 (7.9)	
30–34	1,941,052 (87.7)	272,915 (12.3)	
35–39	699,991 (82.3)	150,716 (17.7)	
≥ 40	153,702 (77.4)	45,001 (22.6)	
Unknown	248,372 (93.0)	18,563 (7.0)	
**Antenatal care visits**			
None	106,694 (92.0)	9,327 (8.0)	*P* < 0.001
1–3	586,349 (93.0)	43,983 (7.0)	
4–6	2,599,192 (93.9)	169,815 (6.1)	
7–9	2,576,046 (90.5)	271,105 (9.5)	
≥ 10	2,480,962 (87.5)	353,423 (12.5)	
Unknown	219,909 (85.5)	37,434 (14.5)	
**Gravidity**			
1	3,440,750 (92.0)	297,971 (8.0)	*P* < 0.001
2–3	4,092,602 (90.7)	421,269 (9.3)	
≥ 4	1,035,800 (86.2)	165,847 (13.8)	
**Parity**			
0	4,848,051 (91.1)	474,646 (8.9)	*P* < 0.001
1	3,209,056 (90.0)	356,733 (10.0)	
≥ 2	512,045 (90.5)	53,708 (9.5)	
**Previous caesarean deliveries**			
0	7,274,553 (91.3)	692,669 (8.7)	*P* < 0.001
1	1,220,781 (87.1)	180,214 (12.9)	
≥ 2	73,818 (85.8)	12,204 (14.2)	
**Foetal presentation**			
Cephalic	8,298,133 (90.8)	839,684 (9.2)	*P* < 0.001
abnormal lies	267,253 (85.8)	44,191 (14.2)	
Unknown	3,766 (75.7)	1,212 (24.3)	
**Large for gestational age**			
No	7,759,647 (91.0)	764,956 (9.0)	*P* < 0.001
Yes	809,505 (87.1)	120,131 (12.9)	
**Delivery method**			
Vaginal	4,944,933 (93.9)	321,071 (6.1)	*P* < 0.001
Caesarean section	3,624,219 (86.5)	564,016 (13.5)	

**Fig. 1:**
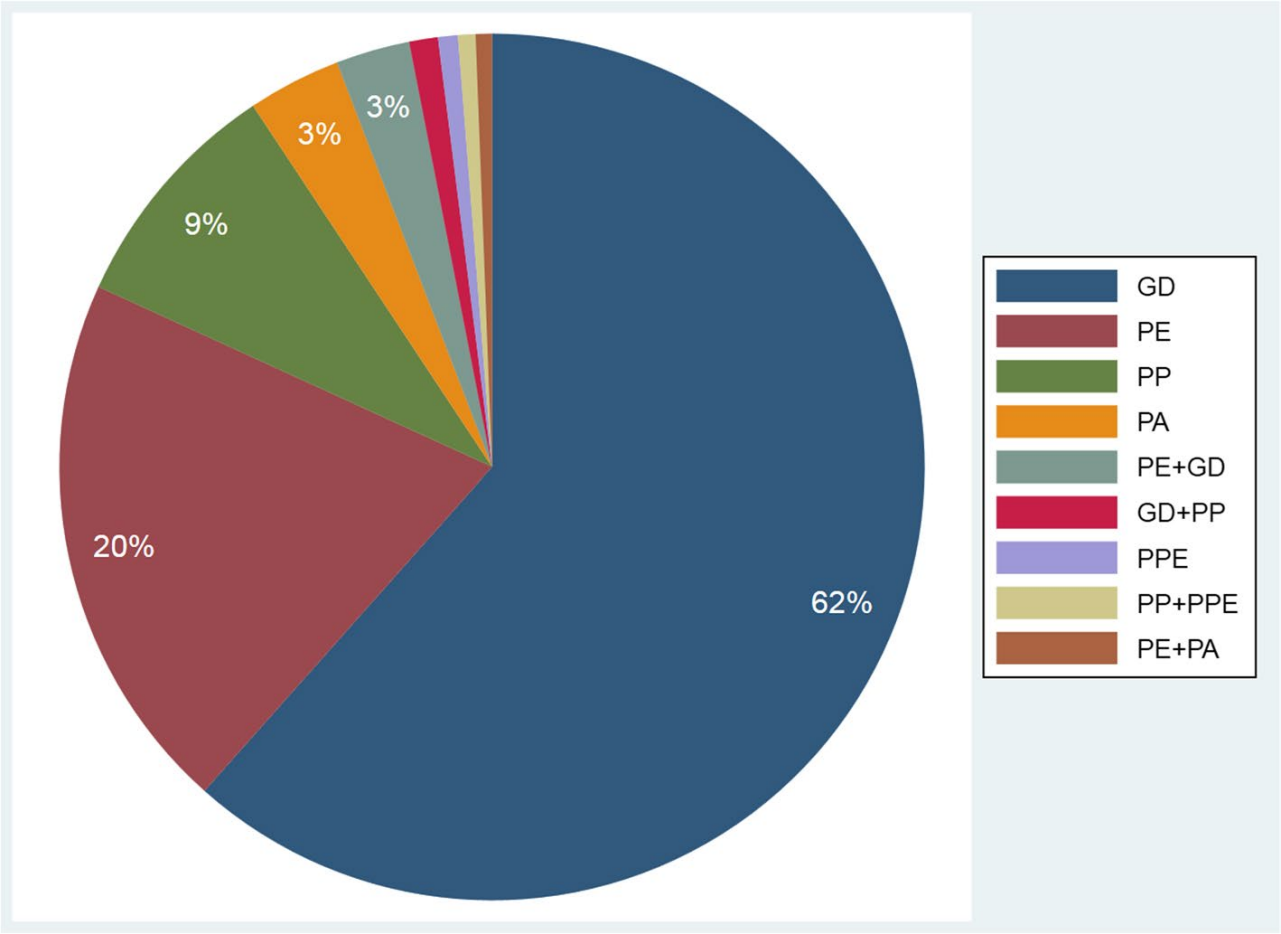
Type and percent of observed pregnancy complications among 875,245 pregnant women. Note: We did show more than 0.3% of observed combinations of pregnancy complications among 885,087 pregnant women. Less than 1% of observed combinations of pregnancy complications were not labelled. *GD*: Gestational diabetes, *PE*: Preeclampsia, *PP*: Placenta praevia, *PA*: Placental abruption, *PPE*: Placenta percreta

Overall, 8.8% of the women had a single pregnancy complication (830,648) and 0.6% had two or more (54,439). Thus, most pregnancy complications occurred as single events (Fig. 1). Among these, gestational diabetes mellitus was the most common pregnancy complication, followed by preeclampsia, placenta previa, placental abruption and placenta percreta.

### Trends over time in UR rates

**Fig. 2:**
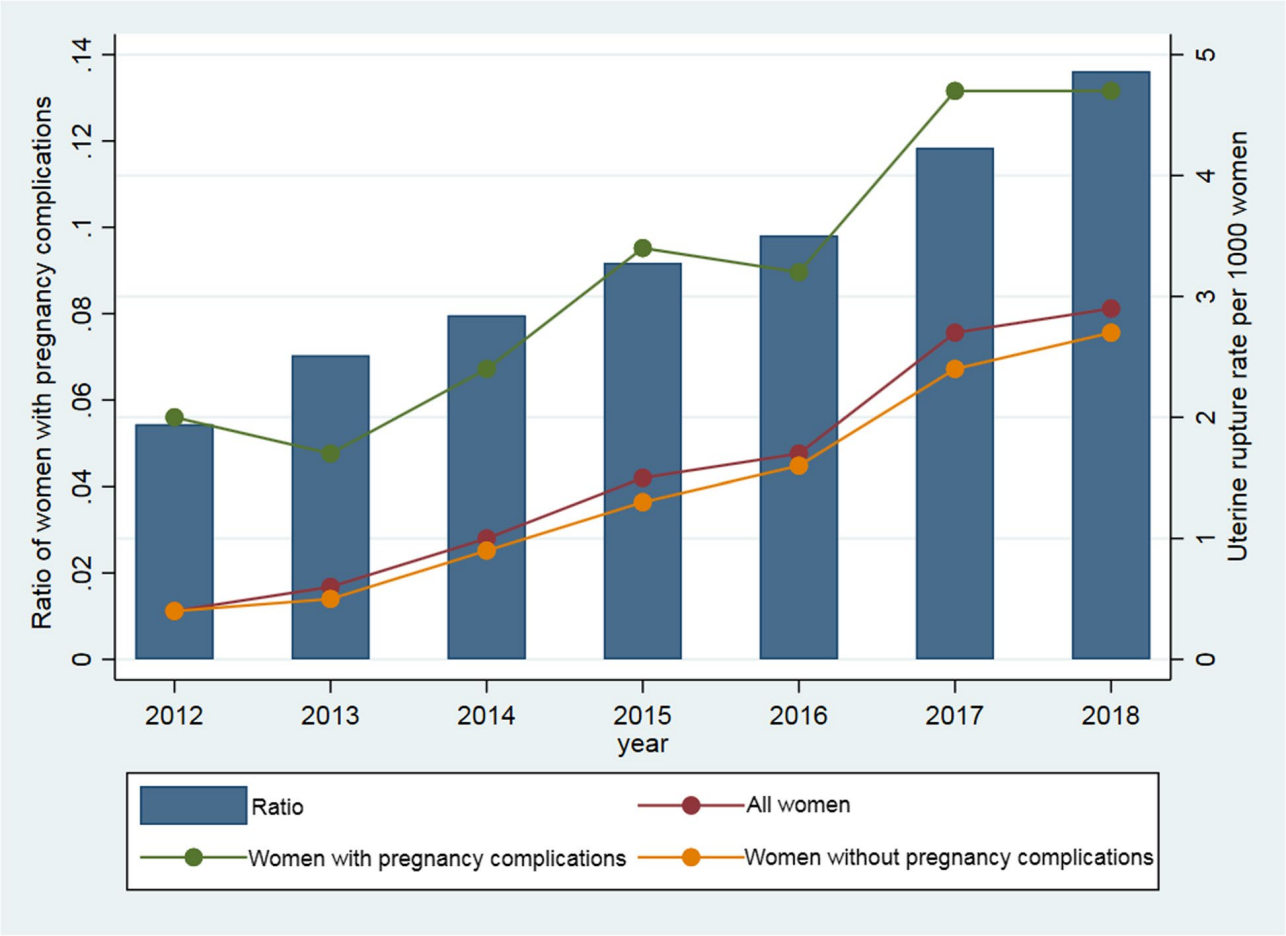
Uterine rupture rate and the ratio of women with pregnancy complications, China, 2012–2018. Note: The UR rate was weighted for the sampling distribution of the population covered by the Chinese National Maternal Near Miss Surveillance System. The UR rate was the number of pregnant women with UR per 1,000 pregnant women. Ratio of women with pregnancy complications was the ratio of women with pregnancy complications to all women

There were 16,949 pregnant women with UR, giving a weighted UR rate of 1.6 cases per thousand pregnant women. Figure 2 shows that the rate of UR was markedly higher in women with pregnancy complications than in women without pregnancy complications, irrespective of medical institution or the pregnant woman's sociodemographic and clinical factors. Moreover, the rate of UR in women with pregnancy complications increased as the ratio of women with pregnancy complications increased between 2012 and 2018. However, there was no change in the UR rate in women with pregnancy complications over time after adjustment for the number of previous caesarean deliveries and all other risk factors (Model 2, aRR: 1.44, 95% CI: 0.91–2.29, Additional file Table S1). Similarly, the UR rate did not change over time in women with each pregnancy complication after adjustment (Additional file Table S1).

### Risk of UR stratified by history of caesarean section

The incidence of UR varied in women with different single-complications (Table 2), with the highest rate in women with placenta percreta. Except for preeclampsia, the other four pregnancy complications were associated with a significantly increased risk of UR after adjustment for risk factors (Table 2). After excluding women with previous caesarean deliveries, these associations were substantially elevated. Among women without previous caesarean delivery, the adjusted risk of UR was 1.41 (95% CI: 1.21–1.65) for women with gestational diabetes mellitus, 5.03 (95% CI: 3.40–7.42) for women with placental abruption, 5.38 (95% CI: 3.76–7.70) for women with placenta previa, and 12.79 (95% CI: 7.69–21.27) for women with placenta percreta (Table 2).

**Table 2:** Risk of uterine rupture with pregnancy complications

**Pregnancy complications**^a^	**No. UR**	**UR rate**^b^	**Adjusted UR rate**^c^	**aRR (95%CI): Model 1**^d^	***P* value**	**aRR (95%CI): Model 2**^e^	***P* value**
**All women**							
None	13,651	1.4	1.4	1 (reference)		1 (reference)	
Preeclampsia	338	1.7	1.5	0.97 (0.76–1.24)	0.825	0.89 (0.70–1.14)	0.358
Gestational diabetes mellitus	1,812	3.2	2.5	1.36 (1.16–1.60)	< 0.001	1.20 (1.03–1.41)	0.020
Placental abruption	175	5.6	5.4	2.44 (1.79–3.33)	< 0.001	2.74 (2.04–3.66)	< 0.001
Placenta previa	520	6.4	3.7	2.13 (1.64–2.77)	< 0.001	1.72 (1.33–2.22)	< 0.001
Placenta percreta	88	13.5	6.2	3.81 (2.48–5.83)	< 0.001	2.64 (1.71–4.07)	< 0.001
**Women without previous caesarean delivery**							
None	1,808	0.2	–	1 (reference)		1 (reference)	
Preeclampsia	46	0.3	–	1.02 (0.69–1.49)	0.938	1.02 (0.69–1.49)	0.933
Gestational diabetes mellitus	199	0.4	–	1.43 (1.22–1.67)	< 0.001	1.41 (1.21–1.65)	< 0.001
Placental abruption	46	1.9	–	4.98 (3.38–7.34)	< 0.001	5.03 (3.40–7.42)	< 0.001
Placenta previa	116	2.1	–	5.36 (3.76–7.66)	< 0.001	5.38 (3.76–7.70)	< 0.001
Placenta percreta	25	6.7	–	12.77 (7.69–21.23)	< 0.001	12.79 (7.69–21.27)	< 0.001

^a^Women with no other four complications in each pregnancy complication group. None: Women with none of the five pregnancy complications.

^b^Weighted UR rate per 1000 women.

^c^Weighted, and previous caesarean deliveries adjusted UR rate per 1000 women.

^d^Model 1: adjusted for sampling distribution of population and clustering of births within hospitals, region, hospital level, the number of antenatal visits, the women’s educational level, maternal age at delivery, parity, foetal presentation, gestational hypertension, chronic hypertension, heart disease, hepatic disease, severe anaemia, infection, thrombophlebitis, renal disease, lung disease, connective tissue disorders.

^e^Model 2: adjusted for Model 1 as well as the number of previous caesarean deliveries (0, 1, ≥ 2) and large for gestational age (yes/no).

The results were similar after restricting the dataset to those women without advanced maternal age and multiple gravidities (Additional file Table S2). When women with offspring having abnormal foetal presentation and a birth weight of more than 4000 g were excluded, the risks of UR with pregnancy complications were largely unchanged (Additional file Table S3).

### Co-occurrence of pregnancy complications and UR risk

Compared with having none of the five pregnancy complications, having two or more complications was associated with a statistically significant almost 1.42–fold risk of UR (Model 1, aRR: 1.88, 95% CI: 1.51–2.34; Model 2, aRR: 1.42, 95% CI:1.14–1.77).

### Risk of UR in different gestational age groups

For a small proportion (14.9%, 2,531 of 16,949) of women with UR, the rupture occurred before term birth. An increased risk of UR before term birth was observed among women with gestational diabetes mellitus, placental abruption and placenta percreta (Table 3). The highest risk of UR was observed among women with placenta percreta at 28 to 33 weeks gestation (Model 2, aRR: 6.21, 95% CI: 3.43–11.24). The risk of UR among women with gestational diabetes mellitus was only observed at 34 to 36 weeks gestation (Model 2, aRR: 1.43, 95% CI: 1.03–1.97). Moreover, the risk of UR among women with placenta previa was only observed at term (Model 2, aRR: 1.41, 95% CI: 1.08–1.86).

**Table 3:** Association between pregnancy complications and uterine rupture by gestational age group

**Pregnancy complications**^a^	**Gestational age group (week)**		
	**Early preterm (28–33)**	**Late preterm (34–36)**	**Term (≥ 37)**
**No. UR**			
None	312	1,430	11,909
Preeclampsia	20	72	246
Gestational diabetes mellitus	41	198	1,573
Placental abruption	40	51	84
Placenta previa	46	138	336
Placenta percreta	16	11	61
**UR rate**^b^ **(Adjusted UR rate**^c^**)**			
None	2.5 (2.5)	3.8 (3.4)	1.3 (1.3)
Preeclampsia	1.0 (0.7)	2.3 (1.7)	1.7 (1.6)
Gestational diabetes mellitus	4.7 (3.4)	6.4 (4.3)	3.0 (2.3)
Placental abruption	6.6 (5.0)	7.5 (5.7)	4.6 (5.5)
Placenta previa	5.9 (3.5)	5.9 (3.0)	6.6 (4.1)
Placenta percreta	46.0 (25.1)	15.7 (6.0)	10.9 (5.1)
**aRR (95%CI): Model 1**^d^			
None	1 (reference)	1.72 (1.29–2.30) ^*^	0.72 (0.57–0.91) ^**^
Preeclampsia	0.25 (0.14–0.44)^*^	0.78 (0.44–1.38)	0.89 (0.61–1.31)
Gestational diabetes mellitus	1.29 (0.96–1.73)	1.84 (1.32–2.57) ^*^	1.00 (0.75–1.33)
Placental abruption	1.69 (1.17–2.46)^**^	2.23 (1.62–3.06)^*^	1.85 (1.38–2.50) ^*^
Placenta previa	1.27 (0.89–1.82)	1.34 (0.89–2.03)	1.91 (1.44–2.53) ^*^
Placenta percreta	7.99 (4.43–14.40)^*^	3.10 (1.65–5.82) ^*^	2.52 (1.73–3.69) ^*^
**aRR (95%CI): Model 2**^b^			
None	1 (reference)	1.47 (1.12–1.92) ^**^	0.67 (0.53–0.85) ^**^
Preeclampsia	0.21 (0.12–0.38)^*^	0.63 (0.36–1.12)	0.77 (0.51–1.14)
Gestational diabetes mellitus	1.13 (0.84–1.53)	1.43 (1.03–1.97) ^***^	0.82 (0.62–1.08)
Placental abruption	1.60 (1.00–2.31)^***^	2.13 (1.55–2.92) ^*^	2.12 (1.60–2.81) ^*^
Placenta previa	1.08 (0.76–1.54)	0.97 (0.67–1.43)	1.41 (1.08–1.86) ^***^
Placenta percreta	6.21 (3.43–11.24)^*^	1.92 (0.99–3.71)	1.60 (1.08–2.38) ^***^

^a^Women with no other four complications in each pregnancy complication group. None: Women with none of the five pregnancy complications.

^b^Weighted UR rate per 1000 women.

^c^Weighted, and previous caesarean deliveries adjusted UR rate per 1000 women.

^d^Model 1: adjusted for sampling distribution of population and clustering of births within hospitals, region, hospital level, the number of antenatal visits, the women’s educational level, maternal age at delivery, parity, foetal presentation, gestational hypertension, chronic hypertension, heart disease, hepatic disease, severe anaemia, infection, thrombophlebitis, renal disease, lung disease, connective tissue disorders.

^e^Model 2: adjusted for Model 1 as well as the number of previous caesarean deliveries (0, 1, ≥ 2) and large for gestational age (yes/no).

^*^ *P* < 0.001; ^**^ P < 0.01; ^***^ P < 0.05.

### Role of large for gestational age

The risk of UR in women with gestational diabetes mellitus without an LGA foetus was 1.18–fold (Model 2, aRR: 1.18, 95% CI: 1.00–1.38), and the risk was slightly larger in women with gestational diabetes mellitus and an LGA foetus (Model 2, aRR: 1.28, 95% CI: 1.09–1.50) (Table 4). Among women at 34–36 weeks gestational age, the association between gestational diabetes mellitus with an LGA foetus and UR was slightly elevated (Model 2, aRR: 1.40, 95% CI: 1.09–1.79) (Table 4).

**Table 4:** The association between gestational diabetes mellitus and uterine rupture

**Gestational diabetes mellitus**^a^	**No. UR**	**UR rate**^b^	**Adjusted UR rate**^c^	**aRR (95%CI): Model 1**^d^	***P* value**	**aRR (95%CI): model 2**^e^	***P* value**
**All women**							
None only	12,138	1.4	1.5	1 (reference)		1 (reference)	
None and LGA	1,513	1.7	1.3	1.02 (0.95–1.11)	0.550	0.96 (0.89–1.04)	0.335
Gestational diabetes mellitus only	1,452	3.0	2.5	1.32 (1.11–1.56)	0.001	1.18 (1.00–1.38)	0.046
Gestational diabetes mellitus and LGA	360	4.1	2.5	1.59 (1.34–1.88)	< 0.001	1.28 (1.09–1.50)	0.003
**Women at 34–36 weeks gestational age**							
None only	1,274	3.7	4.3	1 (reference)		1 (reference)	
None and LGA	156	4.2	3.6	1.02 (0.81–1.30)	0.841	0.92 (0.73–1.15)	0.459
Gestational diabetes mellitus only	140	5.4	4.8	1.00 (0.81–1.23)	0.968	0.92 (0.76–1.12)	0.414
Gestational diabetes mellitus and LGA	58	10.9	6.5	1.77 (1.38–2.26)	< 0.001	1.40 (1.09–1.79)	0.009

^a^Women with no other four complications in gestational diabetes mellitus. None: Women with none of the five pregnancy complications. *LGA*: Large for gestational age.

^b^Weighted UR rate per 1000 women.

^c^Weighted, and previous caesarean deliveries adjusted UR rate per 1000 women.

^d^Model 1: adjusted for sampling distribution of population and clustering of births within hospitals, region, hospital level, the number of antenatal visits, the women’s educational level, maternal age at delivery, parity, foetal presentation, gestational hypertension, chronic hypertension, heart disease, hepatic disease, severe anaemia, infection, thrombophlebitis, renal disease, lung disease, connective tissue disorders.

^e^Model 2: adjusted for Model 1 as well as the number of previous caesarean deliveries (0, 1, ≥ 2).

## Discussion

Using data from more than 9 million singleton pregnant women in China, we found that approximately one-tenth of all women had pregnancy complications, and most occurred as single events. The incidence of UR varied in women with different single-complications, and the highest rate was observed in women with placenta percreta. Gestational diabetes mellitus, placental abruption, placenta previa and placenta percreta were associated with a substantially increased risk of UR, and the risks for UR were 1- to 3-fold higher among women with these pregnancy complications. These associations persisted in women without previous caesarean delivery. Moreover, a significantly increased risk of UR before term birth was observed in women with gestational diabetes mellitus, placental abruption and placenta percreta. The risk of UR was slightly higher in women with gestational diabetes mellitus who had an LGA foetus, especially at 32 to 36 weeks gestation.

In our data, the largest increased risk of UR was seen for placenta percreta (aRR: 2.64, 95% CI: 1.71–4.07). Among women without previous caesarean delivery, the risk of UR was approximately 13 times higher in women with placenta percreta than in those without pregnancy complications (aRR: 12.79, 95% CI: 7.69–21.27). A large retrospective cohort study from Negev found that the risk of UR was increased in women with placenta accreta spectrum disorders (including placenta percreta) (OR: 6.42, 95% CI: 2.0–20.4) [17]. Moreover, previous research found that UR occurs in women without a history of caesarean section [3, 35, 36] and identified spontaneous UR due to placenta percreta as occurring in a primigravida woman without prior uterine operation [37]. Thus, our findings are consistent with previous studies, suggesting that placenta percreta (especially without previous caesarean delivery) may increase the risk of UR.

To date, few studies have reported the association of UR with preeclampsia/eclampsia and diabetes mellitus. However, there are inconsistent conclusions. A population-based Negev study of 138 pregnant women with UR found that hypertension disorders (including preeclampsia/eclampsia) were associated with a twofold increased risk of UR (OR: 2.05, 95% CI: 1.20–3.50), but diabetes mellitus (prepregnancy and gestation) was not related to the risk of UR (OR: 0.87, 95% CI: 0.41–1.86) [7]. However, this study was limited because the diagnoses of hypertension disorder and diabetes mellitus did not distinguish between different subtypes. In contrast, we found that the risk of UR was linked to gestational diabetes mellitus (aRR: 1.20, 95% CI: 1.03–1.41), but not associated with preeclampsia (aRR: 0.89, 95% CI: 0.70–1.14). Our finding is similar to results from two previous studies, where gestational diabetes was associated with increased risk of UR (aOR: 5.78, 95% CI: 1.12–20.00) [19], and eclampsia was not related to the risk of UR (aOR: 0.08, 95% CI: 0.01–0.71) [20].

Furthermore, we found that about six out of 1,000 women had two or more pregnancy complications. A previous study identified placenta previa as often occurring alongside placenta accreta, leading to a higher incidence of bleeding complications [38]. Moreover, placental abruption concurrently presents with preeclampsia in the same pregnancy, and these two complications have a similar pathogenesis, such as placental ischaemia [15]. The cooccurrence of preeclampsia and placental abruption was associated with worse maternal, foetal and neonatal outcomes (e.g., stillbirth/neonatal deaths) [39]. However, the combined effect of pregnancy complications on the risk of UR has been less closely studied. Our results indicate that having two or more pregnancy complications was associated with the risk of UR (Model 2, aRR: 1.42, 95% CI: 1.14–1.77), but we failed to assess the effects of unique combinations due to small samples.

The occurrence of UR may be directly or indirectly caused by a weak myometrium and excessive expansion of the uterine cavity. In our study, we found that placenta percreta was associated with an increased risk of UR in women. The possible reason for this might be the thinning of the uterus after multiple induced abortions, and the placental villi invade the myometrium at the site of placental implantation (particularly at a previous scar site), resulting in UR [18]. In addition, we found that the risk of UR was slightly higher in women with gestational diabetes mellitus who had an LGA foetus (especially at 34–36 weeks gestational age). we hypothesize that excess foetal growth leads to excessive expansion of the uterine cavity during late pregnancy among diabetic women, resulting in UR.

UR often occurs before or during labour without warning. The risk of UR can increase in the presence of predisposing factors. Clinicians may tend to focus on women with a history of caesarean delivery, ignoring those without such a history but with complications during pregnancy. Current guidelines developed by the American College of Obstetricians and Gynaecologists (ACOG) and Royal College of Obstetricians and Gynaecologists (RCOG) place a strong emphasis on the impact of vaginal trial delivery after caesarean section on UR [21, 22]. Unfortunately, there is currently a lack of guidelines for pregnant women with pregnancy complications to prevent the occurrence of UR. For example, current guidelines developed by the ACOG only recommend that women with gestational diabetes mellitus should be counselled regarding the risks and benefits of a scheduled caesarean delivery when the estimated foetal weight is 4,500 g or more [40]. Our findings suggest that controlling weight throughout pregnancy for women with gestational diabetes mellitus may play an important role in preventing the occurrence of UR. Our findings also support the recommendation that women with uncomplicated placenta previa should have a planned delivery at 36–37 weeks of gestation to avoid haemorrhage [41]. Moreover, evidence has shown that an accurate prenatal diagnosis and a standardized multidisciplinary team approach improve the pregnancy outcomes of women with placenta percreta [42]. Thus, standardized protocols for prenatal diagnosis and management of pregnancy complications may help to reduce the occurrence of UR.

Our study has a number of strengths. First, we used common protocols to collect data through uniformly trained clinicians, so the data quality was high. Second, the large sample size allowed us to analyse the risk of UR with pregnancy complications in several subgroups. Third, we were able to adjust for several potential confounders (e.g., the number of previous caesarean deliveries, LGA, abnormal foetal presentation, advanced maternal age, multiple gravidities, coexisting comorbidity, etc.).

We acknowledge some limitations within this study. First, there may be a possibility of the underreporting of pregnancy complications in our retrospective study. Additionally, some women with pregnancy complications may lost to follow up, when they abandoned treatment or were transferred to other non-monitoring hospitals. However, some women with pregnancy complications may also be transferred from other non-monitoring hospitals into monitoring hospitals in the NMNMSS. Second, there was a lack of information on the duration and severity of pregnancy complications in our study, and there may be deviations in evaluating the impact of pregnancy complications on the risk of UR. Third, the only life-threatening UR is the complete UR, but we did not distinguish between complete and partial UR [10]. Meanwhile, the diagnosis of some UR cases simply based on imaging may be inaccurate. Fourth, we were unable to obtain several variables that may have been related to the occurrence of UR, including information on the intended mode of delivery [43], the interval between this pregnancy and the last caesarean section [33], the history of other uterine operations (e.g., myomectomy) [19], and uterine anomalies [44]. Therefore, the estimation of UR risk may be biased.

## Conclusions

Our study identified that the risk of UR is linked to some pregnancy complications (gestational diabetes mellitus, placental abruption, placenta previa and placenta percreta). An increased risk of UR before term birth was observed among women with gestational diabetes mellitus, placental abruption and placenta percreta. The risk of UR was slightly higher in women with gestational diabetes mellitus who had an LGA foetus, especially at 32 to 36 weeks gestation. More research is needed to determine what mechanisms underlie the association between pregnancy complications and UR, and what clinical follow-up and interventions would be most appropriate and effective for women with pregnancy complications.

## Supplementary Information

**Additional file 1:** **Table S1.** Time trends in uterine rupture rates by the presence of pregnancy complications. **Table S2.** Risk of uterine rupture with pregnancy complications restricting women without advanced age (≥35 years) and multiple gravidities (≥4). **Table S3.** Risk of uterine rupture with pregnancy complications restricting women with offspring having a cephalic lie and a birth weight of less than or equal to 4000 g

## Data Availability

The datasets used and/or analyzed during the current study are available from the corresponding author on reasonable request.

## Abbreviations

NMNMSS: National Maternal Near Miss Surveillance System
UR: Uterine rupture
LGA: Large for gestational age
HELLP: Haemolysis, elevated liver enzymes and low platelets
OGTT: Oral glucose tolerance test
aRR: Adjusted relative risk
CI: Confidence Interval
ACOG: American College of Obstetricians and Gynaecologists
RCOG: Royal College of Obstetricians and Gynaecologists
