# Supplementary material for: Pregnancy complications and risk of uterine rupture among women with singleton pregnancies in China
Source: BMC Pregnancy Childbirth. 2022 Feb 16;22:131. doi: 10.1186/s12884-022-04465-w (PMC8851699; doi:10.1186/s12884-022-04465-w)
Supplement: Supplementary file 1 — Additional file 1: Table S1. Time trends in uterine rupture rates by the presence of pregnancy complications. Table S2. Risk of uterine rupture with pregnancy complications restricting women without advanced age (≥35 years) and multiple gravidities (≥4). Table S3. Risk of uterine rupture with pregnancy complications restricting women with offspring having a cephalic lie and a birth weight of less than or equal to 4000 g [file 12884_2022_4465_MOESM1_ESM.docx]

**Pregnancy complications and risk of uterine rupture among women with singleton pregnancies in China**

Jing Tao, Yi Mu, Peiran Chen, Yanxia Xie, Juan Liang, Jun Zhu

**Supplementary Information**

**Table S1 Time trends in uterine rupture rates by the presence of pregnancy complications**

| **Pregnancy complications**^*^ | **2012** | **2013** | **2014** | **2015** | **2016** | **2017** | **2018** |
| --- | --- | --- | --- | --- | --- | --- | --- |
| **No. UR** | | | | | | | |
| All pregnancy complications | 150 | 177 | 312 | 442 | 521 | 867 | 829 |
| Preeclampsia | 22 | 20 | 36 | 56 | 58 | 80 | 66 |
| Gestational diabetes mellitus | 48 | 73 | 140 | 208 | 272 | 540 | 531 |
| Placental abruption | 12 | 17 | 28 | 28 | 23 | 37 | 30 |
| Placenta previa | 36 | 36 | 51 | 92 | 89 | 117 | 99 |
| Placenta percreta | 12 | 3 | 6 | 8 | 15 | 26 | 18 |
| **UR rate^#^ (Adjusted UR rate^##^)** |  |  |  |  |  |  |  |
| All pregnancy complications | 2.0 (3.0) | 1.7 (2.3) | 2.4 (2.9) | 3.4 (3.5) | 3.2 (3.1) | 4.7 (4.0) | 4.7 (4.0) |
| Preeclampsia | 0.9 (0.5) | 0.7 (0.7) | 1.3 (1.0) | 2.1 (1.3) | 1.9 (1.4) | 2.6 (1.9) | 2.6 (2.1) |
| Gestational diabetes mellitus | 1.4 (1.2) | 1.3 (0.8) | 1.8 (1.5) | 2.6 (2.1) | 2.7 (1.7) | 4.6 (2.1) | 4.4 (2.1) |
| Placental abruption | 4.8 (2.4) | 5.2 (2.0) | 6.8 (2.4) | 6.2 (2.8) | 4.5 (2.6) | 6.2 (3.9) | 5.1 (3.7) |
| Placenta previa | 4.2 (5.5) | 3.4 (4.3) | 4.3 (4.7) | 7.8 (7.7) | 6.8 (6.3) | 7.9 (6.8) | 8.8 (7.6) |
| Placenta percreta | 24.0 (31.6) | 5.7 (7.0) | 9.1 (10.2) | 11.6 (12.1) | 15.4 (15.3) | 17.5 (16.1) | 10.8 (9.6) |
| **aRR (95%CI): Model 1^a^** | | | | | | | |
| All pregnancy complications | 1 (reference) | 0.80 (0.61–1.06) | 1.09 (0.77–1.53) | 1.36 (0.95–1.96) | 1.27 (0.79–2.03) | 1.65 (1.07–2.55) ^e^ | 1.64 (1.04–2.60) ^e^ |
| Preeclampsia | 1 (reference) | 0.80 (0.45–1.43) | 1.48 (0.87–2.52) | 2.17 (1.19–3.96) ^e^ | 1.80 (0.84–3.85) | 2.35 (0.99–5.59) | 2.33 (1.16–4.69) ^e^ |
| Gestational diabetes mellitus | 1 (reference) | 0.82 (0.53–1.26) | 1.05 (0.60–1.85) | 1.33 (0.75–2.34) | 1.32 (0.59–2.93) | 1.93 (0.96–3.90) | 1.88 (0.89–3.95) |
| Placental abruption | 1 (reference) | 1.08 (0.47–2.46) | 1.37 (0.71–2.63) | 1.16 (0.54–2.48) | 0.85 (0.40–1.79) | 1.08 (0.51–2.28) | 0.87 (0.40–1.91) |
| Placenta previa | 1 (reference) | 0.80 (0.49–1.31) | 0.96 (0.60–1.54) | 1.71 (0.97–3.00) | 1.45 (0.86–2.44) | 1.65 (1.04–2.61) ^e^ | 1.83 (1.06–3.16) ^e^ |
| Placenta percreta | 1 (reference) | 0.25 (0.67–0.91) | 0.37 (0.17–0.80) ^e^ | 0.44 (0.16–1.24) | 0.64 (0.26–1.61) | 0.77 (0.33–1.80) | 0.48 (0.18–1.24) |
| **aRR (95%CI): Model 2 ^b^** | | | | | | | |
| All pregnancy complications | 1 (reference) | 0.79 (0.60–1.04) | 1.03 (0.74–1.45) | 1.24 (0.87–1.77) | 1.10 (0.70–1.75) | 1.43 (0.93–2.19) | 1.44 (0.91–2.29) |
| Preeclampsia | 1 (reference) | 0.75 (0.42–1.34) | 1.34 (0.78–2.30) | 1.91 (1.06–3.44) ^e^ | 1.51 (0.72–3.20) | 1.92 (0.83–4.42) | 1.96 (0.97–3.94) |
| Gestational diabetes mellitus | 1 (reference) | 0.87 (0.56–1.35) | 1.09 (0.62–1.92) | 1.30 (0.75–2.24) | 1.23 (0.57–2.65) | 1.82 (0.92–3.60) | 1.80 (0.87–3.74) |
| Placental abruption | 1 (reference) | 1.04 (0.46–2.37) | 1.22 (0.64–2.32) | 1.06 (0.50–2.25) | 0.72 (0.34–1.50) | 0.93 (0.45–1.94) | 0.75 (0.34–1.65) |
| Placenta previa | 1 (reference) | 0.79 (0.48–1.29) | 0.90 (0.56–1.43) | 1.50 (0.86–2.63) | 1.24 (0.75–2.05) | 1.39 (0.89–2.18) | 1.55 (0.91–2.64) |
| Placenta percreta | 1 (reference) | 0.24 (0.06–0.92) | 0.35 (0.16–0.77) ^d^ | 0.42 (0.15–1.14) | 0.60 (0.25–1.45) | 0.67 (0.29–1.54) | 0.40 (0.16–1.04) |

^*^ Women with no other four complications in each pregnancy complication group.

# Weighted UR rate per 1000 women

**^##^** Weighted, and previous caesarean deliveries adjusted UR rate per 1000 women

**^a^** Model 1: adjusted for sampling distribution of population and clustering of births within hospitals, region, hospital level, the number of antenatal visits, the women’s educational level, maternal age at delivery, parity, foetal presentation, gestational hypertension, chronic hypertension, heart disease, hepatic disease, severe anaemia, infection, thrombophlebitis, renal disease, lung disease, connective tissue disorders.

**^b^** Model 2: adjusted for Model 1 as well as the number of previous caesarean deliveries (0, 1, ≥2) and large for gestational age (yes/no).

**^c^** *P* < 0.001; ^d^ *P* < 0.01; ^e^ *P* < 0.05

**Table S2 Risk of uterine rupture with pregnancy complications restricting women without advanced age (≥35 years) and multiple gravidities (≥4)**

| **Pregnancy complications**^*^ | **No. UR** | **UR rate^#^** | **Adjusted UR rate^##^** | **aRR (95%CI): Model 1^a^** | ***P* value** | **aRR (95%CI): Model 2^b^** | ***P* value** |
| --- | --- | --- | --- | --- | --- | --- | --- |
| **All women** | | | | | | | |
| None | 7,445 | 1.0 | 1.0 | 1 (reference) |  | 1 (reference) |  |
| Preeclampsia | 168 | 1.3 | 1.2 | 1.16 (0.87–1.54) | 0.306 | 1.00 (0.74–1.32) | 0.941 |
| Gestational diabetes mellitus | 795 | 2.1 | 1.9 | 1.44 (1.22–1.70) | < 0.001 | 1.29 (1.10–1.51) | 0.002 |
| Placental abruption | 89 | 3.8 | 3.8 | 2.51 (1.80–3.51) | < 0.001 | 2.69 (1.95–3.71) | < 0.001 |
| Placenta previa | 237 | 5.3 | 3.5 | 2.79 (2.07–3.77) | < 0.001 | 2.20 (1.61–3.00) | < 0.001 |
| Placenta percreta | 32 | 10.6 | 6.0 | 4.94 (3.01–8.12) | < 0.001 | 3.52 (2.12–5.84) | < 0.001 |
| **Women without previous cesarean delivery** | | | | | | | |
| None | 1,292 | 0.2 | – | 1 (reference) |  | 1 (reference) |  |
| Preeclampsia | 30 | 0.3 | – | 1.06 (0.71–1.59) | 0.782 | 1.06 (0.71–1.58) | 0.781 |
| Gestational diabetes mellitus | 120 | 0.4 | – | 1.41 (1.13–1.77) | 0.003 | 1.39 (1.12–1.74) | 0.003 |
| Placental abruption | 28 | 1.5 | – | 4.93 (3.07–7.91) | < 0.001 | 4.98 (3.09–8.01) | < 0.001 |
| Placenta previa | 69 | 2.1 | – | 6.69 (4.35–10.29) | < 0.001 | 6.72 (4.36–10.35) | < 0.001 |
| Placenta percreta | 13 | 6.1 | – | 15.40 (7.91–30.00) | < 0.001 | 15.41 (7.91–30.04) | < 0.001 |

^*^ Women with no other four complications in each pregnancy complication group. None: Women with none of the five pregnancy complications.

^#^ Weighted UR rate per 1000 women

**^##^** Weighted, and previous caesarean deliveries adjusted UR rate per 1000 women

**^a^** Model 1: adjusted for sampling distribution of population and clustering of births within hospitals, region, hospital level, the number of antenatal visits, the women’s educational level, maternal age at delivery, parity, foetal presentation, gestational hypertension, chronic hypertension, heart disease, hepatic disease, severe anaemia, infection, thrombophlebitis, renal disease, lung disease, connective tissue disorders.

**^b^** Model 2: adjusted for Model 1 as well as the number of previous caesarean deliveries (0, 1, ≥2) and large for gestational age (yes/no).

**Table S3 Risk of uterine rupture with pregnancy complications** **restricting women with** **offspring having a cephalic lie and a birth weight of less than or equal to 4000 g**

| **Pregnancy complications**^*^ | **No. UR** | **UR rate^#^** | **Adjusted UR rate^##^** | **aRR (95%CI): Model 1^a^** | ***P* value** | **aRR (95%CI): Model 2^b^** | ***P* value** |
| --- | --- | --- | --- | --- | --- | --- | --- |
| **All women** | | | | | | | |
| None | 12,571 | 1.4 | 1.4 | 1 (reference) |  | 1 (reference) |  |
| Preeclampsia | 294 | 1.7 | 1.5 | 0.95 (0.74–1.22) | 0.674 | 0.87 (0.67–1.12) | 0.281 |
| Gestational diabetes mellitus | 1,587 | 3.2 | 2.5 | 1.35 (1.15–1.58) | < 0.001 | 1.19 (1.02–1.39) | 0.027 |
| Placental abruption | 159 | 5.5 | 5.4 | 2.45 (1.78–3.37) | < 0.001 | 2.78 (2.07–3.73) | < 0.001 |
| Placenta previa | 448 | 6.3 | 3.7 | 2.15 (1.62–2.85) | < 0.001 | 1.73 (1.31–2.30) | < 0.001 |
| Placenta percreta | 74 | 13.7 | 6.0 | 3.92 (2.56–6.01) | < 0.001 | 2.61 (1.69–4.03) | < 0.001 |
| **Women without previous cesarean delivery** | | | | | | | |
| None | 1,594 | 0.2 | – | 1 (reference) |  | 1 (reference) |  |
| Preeclampsia | 36 | 0.3 | – | 0.97 (0.63–1.48) | 0.876 | 0.97 (0.63–1.48) | 0.880 |
| Gestational diabetes mellitus | 167 | 0.4 | – | 1.40 (1.18–1.66) | < 0.001 | 1.39 (1.18–1.65) | < 0.001 |
| Placental abruption | 38 | 1.7 | – | 4.70 (2.98–7.41) | < 0.001 | 4.71 (2.99–7.44) | < 0.001 |
| Placenta previa | 94 | 2.0 | – | 5.50 (3.70–8.17) | < 0.001 | 5.47 (3.69–8.12) | < 0.001 |
| Placenta percreta | 17 | 5.8 | – | 11.85 (6.56–21.41) | < 0.001 | 11.82 (6.54–21.36) | < 0.001 |

^*^ Women with no other four complications in each pregnancy complication group. None: Women with none of the five pregnancy complications.

^#^ Weighted UR rate per 1000 women

**^##^** Weighted, and previous caesarean deliveries adjusted UR rate per 1000 women

**^a^** Model 1: adjusted for sampling distribution of population and clustering of births within hospitals, region, hospital level, the number of antenatal visits, the women’s educational level, maternal age at delivery, parity, gestational hypertension, chronic hypertension, heart disease, hepatic disease, severe anaemia, infection, thrombophlebitis, renal disease, lung disease, connective tissue disorders.

**^b^** Model 2: adjusted for Model 1 as well as the number of previous caesarean deliveries (0, 1, ≥2) and large for gestational age (yes/no).
